# The Sirtuin-2 Inhibitor AK7 Is Neuroprotective in Models of Parkinson’s Disease but Not Amyotrophic Lateral Sclerosis and Cerebral Ischemia

**DOI:** 10.1371/journal.pone.0116919

**Published:** 2015-01-21

**Authors:** Xiqun Chen, Pauline Wales, Luisa Quinti, Fuxing Zuo, Sébastien Moniot, Fanny Herisson, Nazifa Abdul Rauf, Hua Wang, Richard B. Silverman, Cenk Ayata, Michelle M. Maxwell, Clemens Steegborn, Michael A. Schwarzschild, Tiago F. Outeiro, Aleksey G. Kazantsev

**Affiliations:** 1 Department of Neurology, Massachusetts General Hospital, Harvard Medical School, Boston, Massachusetts, 02129, United States of America; 2 Department of NeuroDegeneration and Restorative Research, Center for Nanoscale Microscopy and Molecular Physiology of the Brain, University Medical Center Goettingen, Waldweg 33, 37073, Goettingen, Germany; 3 Department of Biochemistry, University of Bayreuth, Universitaetsstrasse 30, 95447, Bayreuth, Germany; 4 Department of Chemistry, Department of Molecular Bioscience, Chemistry of Life Processes Institute, Center for Molecular Innovation and Drug Discovery, Northwestern University, Evanston, Illinois, 60208-3113, United States of America; University of Nebraska Medical center, UNITED STATES

## Abstract

Sirtuin deacetylases regulate diverse cellular pathways and influence disease processes. Our previous studies identified the brain-enriched sirtuin-2 (SIRT2) deacetylase as a potential drug target to counteract neurodegeneration. In the present study, we characterize SIRT2 inhibition activity of the brain-permeable compound AK7 and examine the efficacy of this small molecule in models of Parkinson’s disease, amyotrophic lateral sclerosis and cerebral ischemia. Our results demonstrate that AK7 is neuroprotective in models of Parkinson’s disease; it ameliorates alpha-synuclein toxicity *in vitro* and prevents 1-methyl-4-phenyl-1,2,3,6-tetrahydropyridine (MPTP)-induced dopamine depletion and dopaminergic neuron loss *in vivo*. The compound does not show beneficial effects in mouse models of amyotrophic lateral sclerosis and cerebral ischemia. These findings underscore the specificity of protective effects observed here in models of Parkinson’s disease, and previously in Huntington’s disease, and support the development of SIRT2 inhibitors as potential therapeutics for the two neurodegenerative diseases.

## Introduction

Mammalian NAD^+^-dependent sirtuin deacetylases (SIRT1-SIRT7) regulate diverse physiological functions in cells and are implicated as potential modifiers of neurogenerative diseases [1]. The second family member, SIRT2, has been identified as an α-tubulin deacetylase [2]. It has become evident however, that SIRT2 acts on a broad variety of protein substrates implicated in important cellular processes, including transcriptional regulation, cytoskeletal organization, and microtubule dynamics, suggesting a broad regulatory role for this protein that is likely distinct in different cell types [3, 4]. SIRT2 is highly abundant in the adult brain, where its alternatively spliced truncated isoform (SIRT2.2) is preferentially expressed and accumulates with age [5]. High-level of SIRT2 expression is detected in oligodendrocytes [6, 7]. SIRT2 is expressed in neurons as well, although the precise protein function(s) in these cells is still uncertain [5, 8]. Recently, an intronic polymorphism in the SIRT2 gene (SNP rs10410544) has been identified as a risk factor and modifier of Alzheimer’s disease, further illuminating the role of this deacetylase in neurodegeneration [9, 10].

We previously showed that genetic or pharmacological inhibition of SIRT2 is protective in primary neuronal and invertebrate animal models of Parkinson’s disease (PD) [11]. Among the small molecule SIRT2 inhibitors, AK-1, a sulfobenzoic acid derivative, showed consistent protective effects [11]. In addition, brain delivery of AK-1 by an osmotic minipump was safe and neuroprotective in a mouse model of frontotemporal dementia (FTD) based on the expression of mutant tau protein [12]. Protective effects of the AK-1 SIRT2 inhibitor have also been shown in primary neuronal, *C. elegans*, and *Drosophila* models of Huntington’s disease (HD) [8]. Subsequently, we developed a brain-permeable analog of AK-1, the sulfobenzoic acid derivative AK7, and characterized its selective SIRT2 inhibition activity and protective effects in primary HD neurons [13]. Furthermore, treatment with AK7 improved motor function, extended survival, and reduced brain atrophy in two genetic mouse models of HD [14]. Overall, these results suggest that AK7-medicated SIRT2 inhibition counteracts neurodegenerative processes.

In the present study, we characterized the mechanism of action of the brain-permeable SIRT2 inhibitor AK7 and examined its efficacy in cellular aSyn and mouse 1-methyl-4-phenyl-1,2,3,6-tetrahydropyridine (MPTP) models of PD. aSyn and MPTP represent genetic and environmental factors that have been implicated in the etiology of the second most common neurodegenerative disease [15, 16]. In addition, to explore potential general benefits of pharmacological SIRT inhibition in neurological conditions, we also tested efficacy of AK7 in established mouse models of amyotrophic lateral sclerosis (ALS) and cerebral ischemia. Roles of SIRT2 have been proposed but remain to be defined in both ALS and cerebral ischemia [17–19].

## Results

### AK7 inhibits SIRT2 and protects against aSyn toxicity *in vitro*

**Figure 1:**
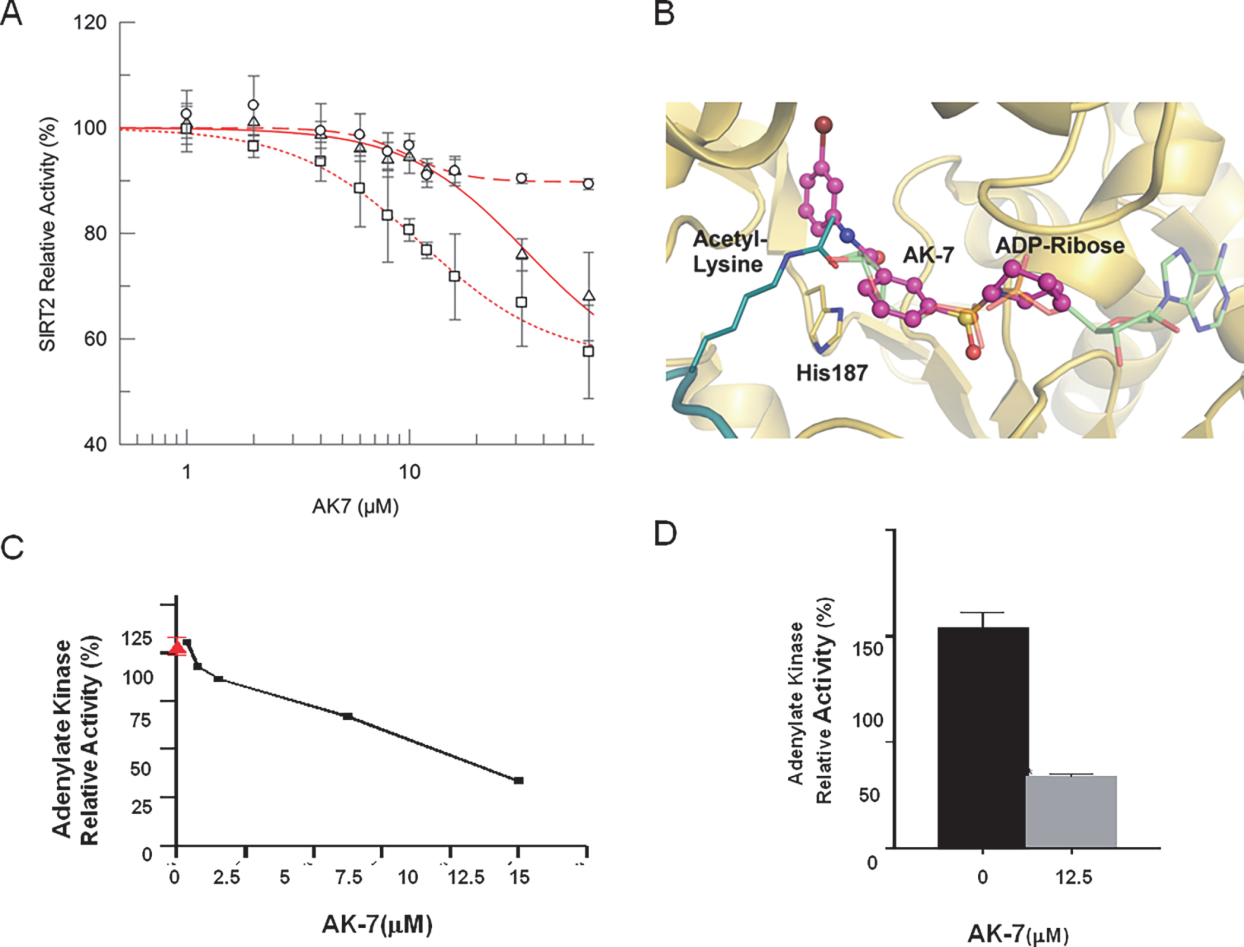
Characterization of the SIRT2 inhibitor AK7 *in vitro* and in a cell model of aSyn toxicity. (A) Determination of AK7 IC_50_ on SIRT2 deacetylase activity at varying substrate (α-tubulin K40 peptide) and co-factor (NAD^+^) concentrations (circles 80 μM α-tubulin/1 mM NAD^+^; squares 80 μM α-tubulin/200 μM NAD^+^; triangles 400 μM α-tubulin/1 mM NAD^+^). Each data point is the average of at least 3 independent measurements. IC_50_ fits are shown as lines. (B) Docking model of a SIRT2/AK7 complex. The structure of SIRT2 (PDB ID 3zgv) is shown in gold with the catalytic His187. ADP-ribose and substrate acetylated lysine are shown in light green and deep teal, respectively. The best pose of AK7 (magenta) mainly occupies the NAD^+^ binding site. (C) LUHMES cells were transduced with a lentivirus encoding aSyn. 10 days after differentiation, in the presence of vehicle alone (red, 100% toxicity) or different concentrations of AK7 (black line), media were collected and AK activity was measured. (D) Percentage of AK activity in the presence of 12.5 M of AK7, compared to vehicle-treated cells (**p<0.01).

Our previous data have shown that treatment with AK7 increases lysine 40 (K40) acetylation of α-tubulin, a well-known substrate of SIRT2, in neuronal cells [13]. To characterize the mechanism by which brain-permeable AK7 inhibits the activity of the SIRT2 deacetylase, we conducted dose-response experiments with recombinant human SIRT2 and acetylated K40- α-tubulin peptide under standard conditions (80 μM peptide/1 mM NAD^+^), which yielded AK7 IC_50_ of 33.8 ± 18.4 M (**Fig. 1A**). Further dose-response experiments were performed in SIRT2 deacetylation reactions with lower NAD^+^ (200 μM) or higher α-tubulin peptide concentration (400 μM), yielding IC_50_ values of 11.4 ± 1.1 μM and 9.8 ± 1.6 μM respectively. The fact that the IC_50_ decreased at lower NAD^+^ concentrations suggests a competition between AK7 and NAD^+^. The behavior with respect to the peptide concentration, however, was less clear. The IC_50_ value was significantly decreased from 33.8 ± 18.4 μM to 9.8±1.6 μM with an increase of peptide concentration, indicating non-competitive binding, i.e. peptide support for compound binding. However, the change of IC_50_ was solely due to an increase of non-inhibited background activity, from ∼55% under standard conditions and low NAD^+^ concentration to ∼90% with high peptide concentration (**Fig. 1A**), but not due to a sideways shift of the transition points. We thus assume that the potency was not directly affected by the peptide, i.e. that SIRT2 inhibition by AK7 is not competitive with the substrate peptide.

Based on the competition results, we generated models for AK7 binding to SIRT2 through docking calculations with SIRT2-peptide complexes. In all three docking procedures (see Experimental procedures) the best pose of AK7 mainly occupies the NAD^+^ binding site of SIRT2 (**Fig. 1B; S1A-B Fig.**), which is consistent with the NAD^+^ competitive mechanism of inhibition. However, slightly varying poses of AK7 were observed. The compound could be observed in both orientations, and slide to different extents into the binding region for the ADP part of NAD^+^. With its terminal ring system, it can either occupy the C-site, which normally accommodates nicotinamide and has been described as an occupancy site for several other sirtuin inhibitors [20, 21] (S1A **Fig.**), or the entry of a large, SIRT2-specific cavity behind the C-site [22] (S1B **Fig.**). In an alternative model, the compound occupancy essentially overlapped with the ADP-ribose part of NAD^+^ (S1B **Fig.**). Any of these positions might explain the NAD^+^ competitive mechanism of SIRT2 inhibition by AK7. Considering its selectivity of SIRT2 inhibition [13], we assume that the compound predominantly exploits the SIRT2-specific active site cavity (**Fig. 1B**). The uncertain interaction of the inhibitor with the peptide might also indicate different AK7 poses in inhibition, depending on the presence of a specific peptide as substrate for deacetylation.

Next, based on our previous study, we confirmed the protective activity of the SIRT2 inhibitor AK7 against aSyn toxicity [11]. Here, we employed a cellular aSyn model of PD in conditionally-immortalized, non-transformed human fetal LUHMES cells differentiated to acquire a dopaminergic neuron-like phenotype under appropriate growth conditions [23, 24]. Overexpression of lentivirus-delivered aSyn in LUHMES cells causes cytotoxicity, which result in two-fold higher release of adenylate kinase (AK) into the culture media, a readout of membrane integrity and cytotoxicity [11]. The effects of AK7 on the viability of LUHMES cells overexpressing aSyn were assessed in a dose-dependent manner (**Fig. 1C**). Dose-dependent protective effects of AK7 were observed, and maximal protection was reached at 12.5 μM (**Fig. 1C-D**). Similar protective effects was achieved using a structurally distinct SIRT2 inhibitor, 3-(benzylthio)-5-[(1-naphthyloxy)methyl]-4-phenyl-4H-1,2,4-triazole (MIND4-11), are shown in S1C-D **Fig.** for comparison.

### AK7 protects against MPTP neurotoxicity in mice

**Figure 2:**
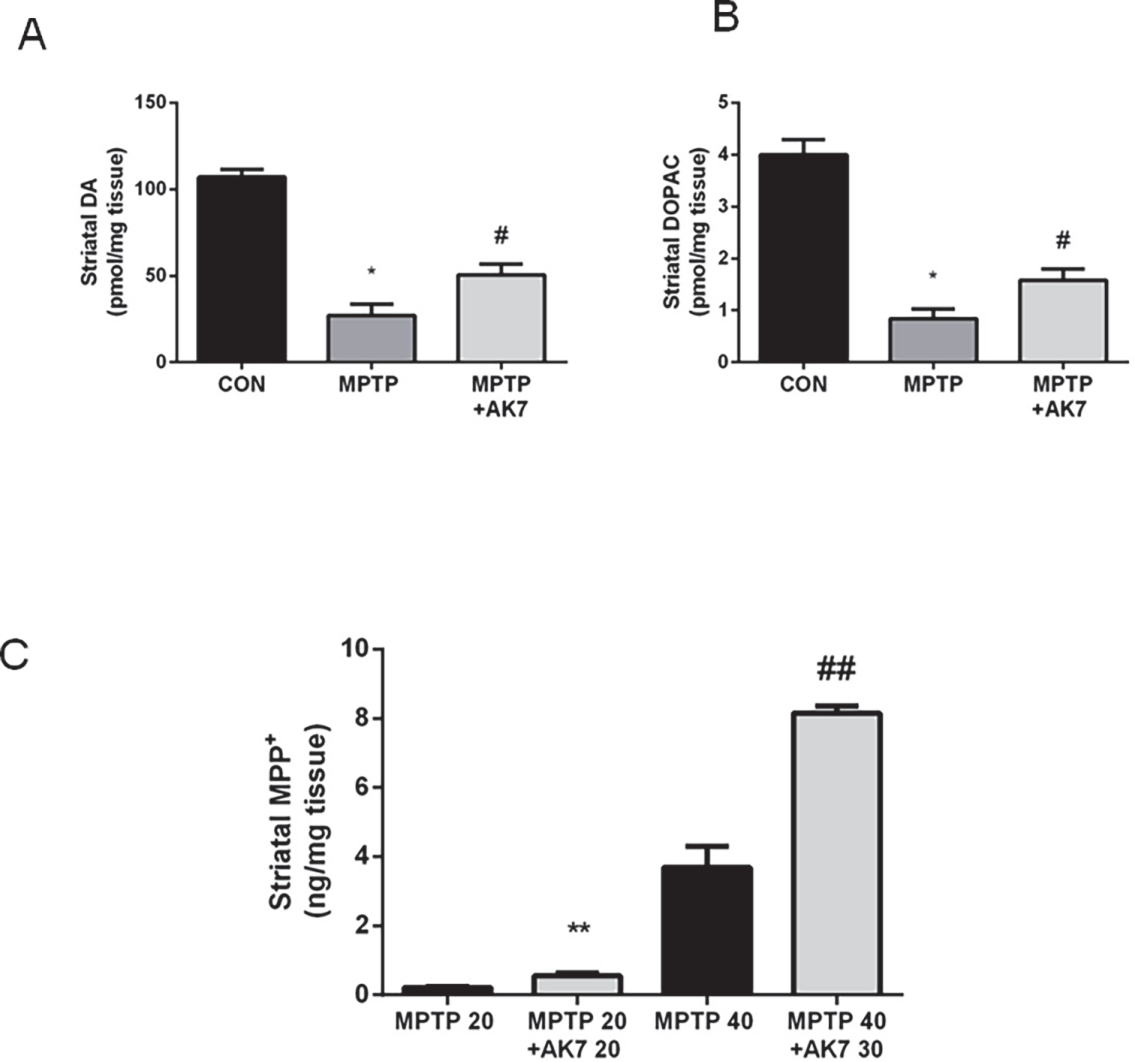
Protective effects of AK7 in acute MPTP mouse model of PD. Mice received a single injection (*i.p.*) of MPTP at 40 mg/kg or saline. AK7 30 mg/kg was injected *i.p.* 10 min before and 50 min after MPTP administration. Animals were sacrificed 7 days after the injection. Striatal DA (A) and metabolite DOPAC (B) were detected by HPLC-ECD (A&B, n = 8–10, *p<0.05 vs CON; # *p*<0.05 vs MPTP). (C) For determination of MPTP metabolism, mice were injected with MPTP and AK7 (10 min before and 50 min after MPTP) at indicated doses and sacrifice 90 min after MPTP treatment. MPP^+^ was detected in the striatum by HPLC (C, n = 5–6, ** ##, *p*<0.01 vs corresponding MPTP control groups).

**Figure 3:**
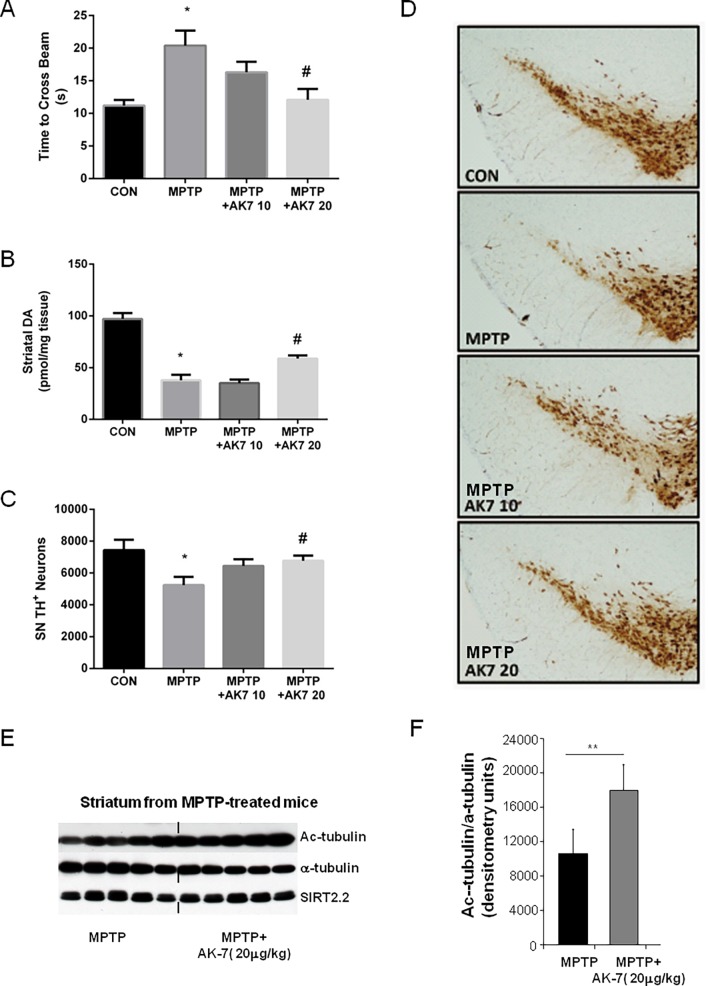
Protective effects of AK7 in subacute MPTP mouse model of PD. Mice were treated with MPTP *i.p.* at 20 mg/kg once daily for 4 days and sacrificed 5 days after the last injection. AK7 was injected at 10 or 20 mg/kg *i.p.* 10 min before and 50 min after MPTP administration. (A) Beam test was performed 3 days after the last MPTP administration. (B) Striatal DA was detected by HPLC-ECD. TH immunohistochemistry was performed and nigral dopaminergic neurons were counted by stereological analysis of TH positive neurons (C and D). A separate experiment was performed using the same treatment regimen but mice were sacrificed 24 hr after the last MPTP injection. Acetylated α-tubulin (Ac-tubulin), total α-tubulin, and brain-predominant SIRT2.2 isofrom was detected in the striatum by Western Blotting (E) and the blot was quantified using Image J (F). (*p<0.05 vs CON; # *p*<0.05 vs MPTP; ** *p*<0.01 vs MPTP).

We then examined the neuroprotective effects of AK7 in *in vivo* MPTP model of PD [15, 25] (**Figs. 2 and 3**). In the acute MPTP paradigm, in which animals were injected *i.p.* once with MPTP 40 mg/kg, and with AK7 at 30 mg/kg 10 min before and 50 min after MPTP injection. AK7 rescued MPTP-induced loss of dopamine (DA) and of the metabolite dihydroxyphenylacetic acid (DOPAC) in the striatum **(Fig. 2A, B)**. AK7, however, appeared to alter MPTP metabolism in the acute setting. Increased levels of MPP^+^, the active, toxic metabolite of MPTP, were detected in the striatum of AK7 treated animals 90 min after MPTP injection (**Fig. 2C**). Thus, AK7 might have even stronger neuroprotective effects to overcome neurotoxicity of increased MPP^+^ in the acute paradigm.

In the subacute paradigm (**Fig. 3**), AK7, when administrated *i.p.* at 20 mg/kg 10 min before and 50min after MPTP 20 mg/kg i.p. once daily for 4 days, improved beam performance 3 days after the last MPTP injection (**Fig. 3A**). High performance liquid chromatography (HPLC) coupled with electrochemical detection (ECD) showed that AK7 at 20 mg/kg attenuated DA loss induced by MPTP in the striatum (**Fig. 3B**). Dopaminergic cell counts in the substantia nigra (SN) demonstrated preservation of dopaminergic neurons in AK7 + MPTP treated mice compared to mice treated with MPTP alone (**Fig. 3C**), as illustrated in Fig. 3D showing tyrosine hydroxylase (TH) -immunostained dopaminergic neurons in the SN. Significant increase of α-tubulin aectylation in the striatum after AK7 treatment was displayed by Western Blotting, confirming compound brain bioactivity of SIRT inhibition; the expression of SIRT2 protein itself (isoform SIRT2.2) was unchanged, as expected (**Fig. 3E, F**).

### Effects of AK7 in a mouse models of ALS

**Figure 4:**
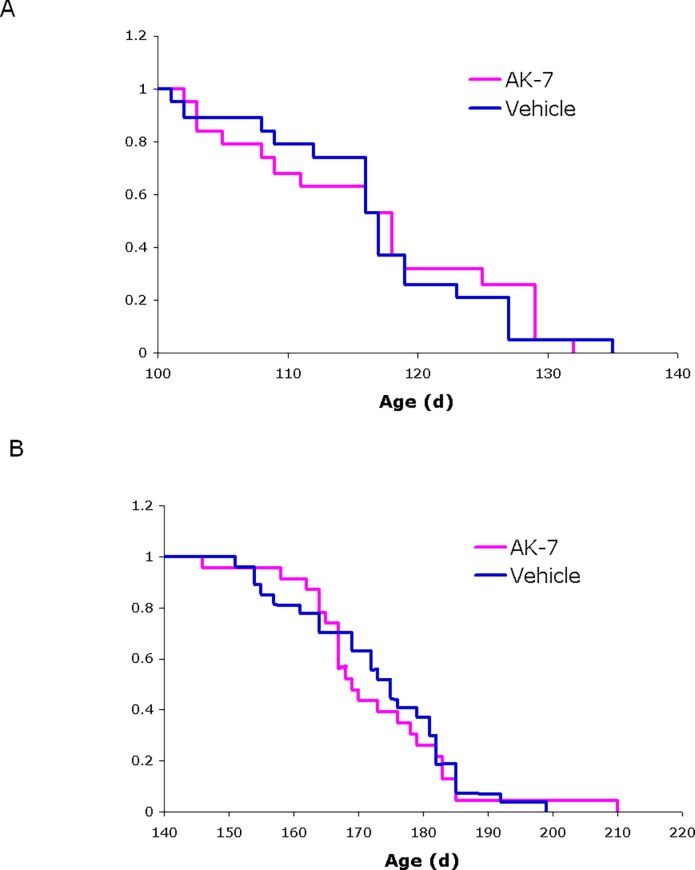
Effects of AK7 in SOD1-G93A mouse model of ALS. Kaplan-Meier probability curves show no significant effects of AK7 treatment on (**A**) symptom onset (118 ± 10.0 days for AK7, 117 ± 8.6 days for vehicle-treated controls) or (**B**) survival (169 ± 12.7 days for AK7, 175 ± 12.4 days for controls) of SOD1-G93A mice in this study. Values are median age ± SD; n = 23 for AK7, n = 27 for vehicle-treated controls.

In a parallel study, we evaluated the effects of AK7 on disease onset and survival in a mouse model of ALS. These studies were conducted using SOD1-G93A transgenic mice (strain B6.Cg-Tg (SOD1*G93A)1Gur/J), which express an ALS-linked human mutant allele of SOD1 [26] and are the *in vivo* model of choice for preclinical efficacy studies in ALS [27, 28]. We observed no significant effect on disease onset or survival in these animals following chronic treatment with AK7 (**Fig. 4**). These results are in accord with a recent report that genetic ablation of SIRT2 does not alter disease progression in ALS mice [29].

### Effects of AK7 in a mouse model of ischemic stroke

A role for SIRT2 in mediating programmed necrosis, and a possible amelioration of necrotic injuries, including those that result from ischemic stroke and myocardial infarction, by inhibition of SIRT2 enzyme activity has been proposed but remains controversial [18, 19]. We examined the effects of AK7 in an experimental mouse model of cerebral ischemia. We did not detect a significant difference between vehicle and AK7-treated groups in mortality, infarct volumes, ischemic tissue swelling, or neurological deficit scores (**Table 1**).

**Table 1:** Effects of AK7 in a mouse model of ischemic stoke.

	**Vehicle (n = 9)**	**AK7 (n = 10)**	***p***
**Mortality (mice)**	1/9	2/10	>0.05
**infarct volumes (mm^3^)**	76 ± 26	85 ± 28	>0.05
**ischemic tissue swelling (mm^3^)**	6 ± 8	9 ± 8	>0.05
**neurological deficit scores (median [interquartile range]**	2.0 [1.0–2.3]	1.5 [1.0–2.0]	>0.05

## Discussion

The present study characterized the SIRT2 inhibition property of the previously identified small molecule AK7 [17] and its effects in models of three neurodegenerative conditions: PD, ALS, and ischemic stroke. Our results demonstrated that AK7 is neuroprotective only in models of PD. Treatment with AK7 protects dopaminergic neurons against aSyn-induced neurotoxicity in differentiated LUHMES cells; in MPTP mouse model, systemic administration of AK7 prevents DA loss, promotes long-term survival of dopaminergic neurons, and preserves functional performance.

The observation that AK7 is neuroprotective in aSyn overexpressing LUHMES cells is consistent with our previous report [11]. The protective effects of AK7 in MPTP mouse model of PD are less expected, since this neurotoxin interferes with mitochondrial complex I. AK7 shows high *in vitro* selectivity for SIRT2, and accordingly increases α-tubulin acetylation (used here as a pharmacodynamic marker for compound activity *in vivo*) in the striatum of MPTP treated mice. It remains to be determined how AK7-mediated SIRT2 inhibition protects against both the genetic and environmental factors in PD. Most likely the beneficial influence of SIRT2 inhibition arises from AK7’s effects on multiple downstream targets/pathways, which are beginning to emerge [4]. The protective actions of AK7 counteracting MPTP toxicity might also be related to aSyn, as implicated by previous studies [30, 31], which would suggest aSyn as a point of convergence of SIRT2-mediated protective pathways in the case of PD.

AK7 treatment increases the concentration of MPP^+^, an active neurotoxic form of MPTP, in the striatum at the peak time after MPTP systemic administration [25], suggesting that the protective effects of SIRT2 inhibition could be underestimated in this model. In initial assessment of possible mechanisms of altered MPTP metabolism, we found no evidence that monoamine oxidase B (MOA-B) that is responsible for the metabolic conversion of MPTP to MPP^+^ is a substrate for SIRT2 deacetylase [4]. Despite SIRT2 selectivity of AK7 *in vitro*, we cannot exclude that the compound may interact with other structurally similar proteins when administered *in vivo*, although whether and how such off-target activity contributes to the altered MPTP metabolism are utterly unknown.

The small, brain-permeable compound AK7 mediates neuroprotection in neuronal and mouse models of PD and HD, indicating common underlying mechanisms in different neurodegenerative diseases and general benefits of pharmacological SIRT2 inhibition [1]. These data suggest that SIRT2 inhibition may stimulate broad neuroprotective responses, downstream from the specific pathological changes that occur in each neurodegenerative condition. Such protective beneficial responses, for example, could be broadly specific to stimulation of microtubule-dependent trafficking or global transcriptional activity.

However, in contrast to pharmacological treatment, no changes in neurological phenotype in the fragment HD mouse model R6/2 were evident in SIRT2 KO genetic background [14, 32]. There were no effects of AK7 treatment in the SOD1-G93A mouse model of ALS, consistent with previously reported negative results of SIRT2 genetic knockout in these mice [29]. Although our preclinical efficacy test of AK7 in a mouse model of ALS yielded negative results, this finding is consistent with the less defined and potentially conflicting roles of SIRT2 in ALS [17]. Moreover, treatment with AK7 was not beneficial in mouse model of stroke, where a therapeutic role of SIRT2 inhibition has been proposed but remains controversial [18, 19]. These negative results suggest specificity of the neuroprotective effects mediated by AK7 in Parkinson’s and Huntington’s disease models and underscore the significance of these effects as observed in this and previous studies [14].

In light of previous studies, the present investigation confirms a critical role for the SIRT2 pathway in PD pathophysiology, where the benefits of SIRT2 inhibition have been observed in diverse models and the efficacy achieved has been most consistent [11, 33]. This specificity further strengthens and validates the therapetutic rationale and provides a mechanistic basis for clinical development of brain-penetrant SIRT2 inhibitors as candidate neuroprotectants for PD, HD, and possibly other related neurodegenerative diseases.

## Materials and Methods

### Biochemical characterization of SIRT2 inhibition property of AK7

**SIRT2 protein preparation and deacetylation assays.** The human SIRT2 catalytic domain (residues 43–356) was cloned into pET-SUMO using Nde1 and Xho1. SIRT2 was expressed using autoinduction in *E. coli* Codon+ as previously described [22]. Harvested cells were resuspended in 50 mM Tris pH 8.0, 500 mM NaCl, 5% glycerol, disrupted using an EmulsiFlex C3 homogenizer (Avestin) and cell debris removed by 45 min centrifugation at 4°C and 20,000 rpm in a HFA22.50 rotor. An affinity chromatography was performed using a HisTrap column (GE Healthcare) and SIRT2 eluted with buffer supplemented with 250 mM imidazol. The fusion protein was digested overnight at 4°C using Sumo-protease and subjected to a reverse affinity purification step. SIRT2 was finally ran over a Superdex 200 gel filtration column (GE healthcare) in 20 mM Tris pH 8.0, 150 mM NaCl, 1 mM TCEP, analyzed by SDS –PAGE, concentrated, and kept at 4°C.

Deacetylase activity assays were performed as described [34]. Briefly, the reaction mix contained 0.8 μM Sirt2 (43–356), 200 μM or 1 mM NAD+ and 80 or 400 μM α-tubulin peptide, MPSD(ac)KTIG (GL Biochem) and AK7 concentrations from 0 to 64 μM with constant 5% DMSO in a 20 mM Na-phosphate buffer at pH 7.5. The reaction was started by adding human recombinant SIRT2 and followed for 45min at 340 nm in a MQX200 (MWG-Biotech) microplate reader. The background signal was measured under similar conditions omitting the substrate peptide from the reaction. Results are the average of at least four measurements and IC_50_ values were determined using Grafit 7 (Erathicus Software).

**Docking of AK7 to the SIRT2 active site.** For generating SIRT2/AK7 models, the compound was docked using the program FlexX (BioSolveIT) and different SIRT2 conformations: The SIRT2 complex with ADP-ribose (pdb ID 3zgv; ligand omitted for the calculation) [22] (Fig. 1B), the SIRT2 complex with a macrocyclic inhibitor peptide (4l3o) [35] (S1A Fig.), or a model of an acetyl-lysine bound SIRT2 generated using 3zgv for the protein and the macrocyclic peptide from 4l3o for the acetyl lysine (S1B Fig.). In all cases the best pose was visualized in the receptor using PyMol (Schrödinger LLC).

### Drug-treatment in viability assay in differentiated LUHMES cells overexpressing aSyn

LUHMES cells, which were a kind gift from Dr. Marcel Leist, University of Konstanz, Germany [23] were maintained as proliferating cultures and differentiated into post-mitotic neuron-like cells on Nunclon plates and flasks, pre-coated with 50 g/mL poly-L-ornithine (Sigma) and 1 g/mL fibronectin (Sigma), as previously described [23, 24]. Briefly, 8×106 proliferating cells were seeded into a T175 flask containing proliferation medium, consisting of advanced DEMEM/F12 (Invitrogen), 2 mM L-glutamine (Sigma-Aldrich), 1× N2 supplement (Invitrogen) and 40 ng/mL recombinant human bFGF (R&D Systems). After 24 hours, proliferation medium was replaced with differentiation medium, composed of advanced DMEM/F12 (Invitrogen), 2 mM L-glutamine (Sigma-Aldrich), 1× N2 supplement (Invitrogen), 1 mM dibutyryl 3’, 5’-cyclic adenosine monophosphate (Sigma-Aldrich), 2.25 M tetracycline and 2 ng/mL recombinant human GDNF (R&D Systems). 48 hr later, cells were trypsinized and seeded into 24-well plates, containing 1mL of differentiation medium and 250,000 cells/well.

**Lenti-virus based expression of aSyn transfer plasmids.** Full-length human aSyn cDNA was subcloned into a second-generation of lentiviral vector pWPI (Tronolab, Switzerland), followed by an IRES-EGFP sequence. The original promoter (EF1) was replaced by the chicken/β-actin promoter. The vector including only the IRES-GFP cassette was used for control experiments. The correct nature of all cloned sequences was confirmed by automated sequencing (StarSeq, Mainz Germany). For lentiviral shRNA production a third generation lentiviral vector pLKO.1 puro (from Sigma Aldrich) containing the following sequence 5’-ACCAAAGAGCAAGTGACAAAT-3’ was used to knock down the gene expression for human SNCA. Control experiments were performed with the vector pLKO.1 puro containing the scrambled sequence 5’-CCTAAGGTTAAGTCGCCCTCG-3’. Second-generation lentiviral particles were generated as described previously [37]. After purification of the modified transfer-vectors and cotransfection with the packaging vectors (Tronolab, Switzerland) into LUHMES cells (Invitrogen) for 48 hr, the supernatant was collected, concentrated by PEG-it Virus Precipitation Solution (System Biosciences) and resuspended in Panserin 401 (PAN, Germany). The measurement of transgene expression has been determined by qRT PCR using SYBR GREEN, and specific primers to the woodchuck hepatitis virus post transcriptional regulatory element (WPRE) as described previously [36]. Viruses were used equimolarly in all applications, stored at −80°C, and kept on ice during cell culture procedures. Transduction was accomplished by incubating undifferentiated LUHMES cells with virus-containing supernatant for 48 hr. GFP-positive cells were selected via FACS sorting (BD Aria II). Viruses were used equimolarly in all applications, stored at −80°C, and kept on ice during cell culture procedures.

**Cell viability assay.** Cell viability was measured by cellular release of adenylate kinase (AK) using quantitative bioluminescent cytotoxicity assay (ToxiLight BioAssay (Lonza) according to the manufacturer’s protocol. 72 hr after cells were seeded into 24-well plates, 500 L of conditioned supernatants were replaced with fresh differentiation medium, containing each of the compounds or vehicle control (DMSO). 96 hr after that, 20 L of cell culture supernatants were added to individual wells of a black-walled, clear-bottom, 96-well microtiter plate. Next, 100 L of ToxiLight AK reagent was added to each well and incubated at room temperature for 5 min. Luminescence was measured, using an Infinte M200 PRO (Tecan) plate reader, and luminescence results of the test wells were expressed as percentage of the control wells.

### Drug trial MPTP mouse model of PD

**Animals and treatment regimens.** Male C57BL/6 mice (∼ 25 g) from the Jackson Laboratories were housed in temperature- and humidity- controlled rooms with a 12-hr dark: light cycle and had free access to food and water. In an acute intoxication paradigm, AK7 was administered *i.p.* 10min before and 50 min after a single dose of 40 mg/kg MPTP-HCl *i.p.* In subacute toxin model, mice were injected with 20 mg/kg MPTP-HCl *i.p.* once daily for 4 days, AK7 was administered *i.p.* at indicated doses 10 min before and 50 min after each MPTP injection. Control animals received vehicle (25% cremophor in PBS) injection. The injection times were determined based on metabolism of both AK7 and MPTP in mouse brain [13, 25].

**Beam test.** For mice that were treated with subacute MPTP regimen, three days after the last MPTP injection, animals were placed on an increasingly narrower beam and total number of steps and time to traverse were recorded. All animals were trained before treatment started [37].

**Measurement of DA and metabolite.** Mice were sacrificed 7 days after a single dose of MPTP in acute regimen and 5 days after the last MPTP injection in subacute regimen by rapid cervical dislocation, and their striata were dissected. DA and its metabolite DOPAC were determined HPLC coupled with ECD [38].

**TH immunohistochemistry and stereological quantification.** Dopaminergic neuron marker TH immunostaining was performed using mouse anti-TH antibody (Sigma, ST. Louis, MO) at 1:1000. Total numbers of TH positive neurons in the SN were counted under blinded conditions using the Bioquant Image Analysis System (R&M Biometrics, Nashville, TN) [38].

**Western blot.** To detect Ac α-tubulin, α-tubulin, and SIRT2.2 in the striatum, mice were treated with subchornic MPTP regimen and were sacrifice 24 hr after the last MPTP injection. Fresh frozen striatal tissue samples were homogenized and proteins were extracted as previously described [5]. Immunoblotting was performed using the following primary antibodies: rabbit anti-SIRT2 (S8447, Sigma-Aldrich), mouse anti-GAPDH (clone 6C5, MAB374, Millipore), mouse anti-α-tubulin (T6074, Sigma-Aldrich), mouse anti-acetylated α-tubulin (clone 611B-1, T6793, Sigma-Aldrich), and rabbit anti-actin (A2066, Sigma-Aldrich). Secondary antibodies were horseradish peroxidase-conjugated anti-rabbit or anti-mouse IgG (Sigma-Aldrich). Band intensity was quantified using ImageJ [5].

### Drug trial in the mutant SOD1-G93A mouse model of ALS

Transgenic ALS model mice used in this study carry the human G93A mutant allele of SOD1 [26] on the C57BL/6 background [39]. SOD1-G93A mice on the C57BL/6 background exhibit delayed onset of clinical symptoms and extended lifespan as compared to the commonly used B6SJL hybrid strain [40], although the overall duration of disease is unchanged. Study animals were generated by backcrossing male B6.Cg-Tg (SOD1*G93A)1Gur/J mice (obtained from The Jackson Laboratory, Bar Harbor, ME) to C57BL/6J females. Mice were genotyped by PCR of tail-tip DNA and housed under standard conditions with free access to food and water.

For drug studies, SOD1-G93A transgenic animals from the same backcross generation were randomly assigned to treatment groups at 55 days of age. Both males are females were used, and sexes and littermates were balanced across treatment groups. AK7 (20 mg/kg) or vehicle (5% Cremaphor in PBS) was administered daily via *i.p.* injection beginning at 60 days of age, before time of disease onset, which is 120 days of age. Mice were weighed and assessed twice weekly for the first signs of disease onset using a standard neurological scoring system for ALS mice. Onset of symptoms was determined by the first appearance of neurological deficit, defined as the inability of the animal to fully splay its hindlimbs when briefly held suspended by its tail. Survival is defined as age at death or euthanasia due to end-stage disease. An animal at end-stage disease is euthanized when it cannot 1) right itself within 15 seconds of being placed on its side, 2) groom its face (detected by the development of infections in one or both eyes), or 3) move around to reach food placed on the cage floor. Time of disease onset and survival were compared among treatment groups using Kaplan-Meier curves and the Log-Rank test.

### Drug trial in an experimental mouse model of cerebral ischemia

Wild type mice (8–10 week-old male C57B6/J; Charles River Labs) were treated with AK7 (30 mg/kg *i.p.*, diluted in 5% cremophor in PBS) or vehicle once at reperfusion (post-ischemic cohort). In a separate cohort, mice were treated twice a day for two days before stroke induction, and then received a dose immediately before the stroke and an additional dose in the evening (peri-ischemic cohort). Because outcomes did not vary between post-ischemic and peri-ischemic treatment paradigms, the data were pooled. Mice were anesthetized by isoflurane (3% induction, 2% maintenance in 30% O_2_, 70% N_2_O), and rectal temperature maintained between 36–36.5°C (FHC, ME, USA). Cerebral blood flow was continuously monitored over the parietal cortex by laser Doppler during the entire procedure. After midline incision, the right carotid artery bifurcation was dissected and external carotid ligated. A clip was then placed on the internal carotid artery, common carotid temporarily ligated. A commercial silicon coated filament (7019, Doccol Corp, MA, USA) was then inserted through the external into the internal carotid artery, and advanced to the middle cerebral artery origin, occlusion of which was confirmed by cerebral blood flow reduction to less than 20% of baseline. The filament was gently pulled out after 1 hour and common carotid ligation released. Successful reperfusion was confirmed by restoration of cerebral blood flow. Mice were placed in a temperature-controlled incubator with easy access to food and water, and body weight was monitored. Neurological outcomes were scored 24 hr after stroke using the following grading system: no neurological deficit (0), forepaw extension deficit during clasping reflex (1), circling behavior (2), comatose (3) and death (4). Mice were euthanized 24 hr after stroke onset with a lethal dose of isoflurane followed by decapitation. Infarct volume was assessed in a blinded fashion by integrating the infarct area in ten 1-mm-thick coronal sections soaked in 2% 2,3,5-triphenyltetrazolium chloride (Sigma, St. Louise, MO) for 15 min protected from light. Infarct volume was calculated by subtracting the volume of ipsilateral non-infarcted tissue from the contralateral hemisphere. Ischemic swelling volume was calculated by subtracting the volume of contralateral hemisphere from the volume of ipsilateral hemisphere.

### Ethics statements

The animal experiments were carried out in strict accordance with the recommendations in the Guide for the Care and Use of Laboratory Animals of the National Institutes of Health. The protocol was approved by the Massachusetts General Hospital Animal Care and Use Committee (approval number 2006N000120) or co-authors’ institutions. Animals were sacrificed by rapid cervical dislocation or euthanized by isoflurane. All efforts were made to minimize suffering.

### Statistical analysis

All values are expressed as mean ± SEM. The difference between two groups was analyzed by the *t* test. Multiple comparisons among groups were performed by one-way analysis of variance and Tukey post hoc analyses. *p<0.05* was considered statistically significant.

## Supporting Information

S1 Fig Docking models of a SIRT2/AK7 complex. The structure of SIRT2 (PDB ID 3zgv) is shown in gold with the catalytic His187. ADP-ribose (3zgv) and substrate acetylated lysine are shown in light green and deep teal, respectively. AK7 was docked either to Sirt2 in complex with a macrocyclic inhibitor peptide (PDB ID 4l3o) (1A) or to a model of an acetyl-lysine bound SIRT2 generated using 3zgv for the protein and the macrocyclic peptide from 4l3o for the acetyl lysine model (1B). In each cases, the best pose of AK7 (magenta) mainly occupies the NAD^+^ binding site. (C, D) Protective effects against aSyn toxicity in LUHMES cells of novel SIRT2 inhibitor MIND4-11. MIND4-11 has been identified as selective SIRT2 inhibitor of improved potency (IC50~5mM), albeit lacking brain-permeable property. (C) LUHMES cells were transduced with a lentivirus encoding aSyn. 10 days after differentiation, in the presence of vehicle alone (red, 100% toxicity) or different concentrations of MIND4-11 (black line), media were collected and AK activity was measured. (D) Percentage of AK activity in the presence of 12.5 M of MIND4-11, compared to vehicle-treated cells (*** *p*< 0.001).
(TIF)

## References

[1] Donmez G, Outeiro TF (2013) SIRT1 and SIRT2: emerging targets in neurodegeneration. EMBO Mol Med 5(3):344–52. 10.1002/emmm.201302451 23417962 PMC3598076

[2] North BJ, Marshall BL, Borra MT, Denu JM, Verdin E (2003) The human Sir2 ortholog, SIRT2, is an NAD+-dependent tubulin deacetylase. Mol Cell 11(2):437–44. 12620231 10.1016/s1097-2765(03)00038-8

[3] Taylor DM, Maxwell MM, Luthi-Carter R, Kazantsev AG (2008) Biological and potential therapeutic roles of sirtuin deacetylases. Cell Mol Life Sci 65(24):4000–18. 10.1007/s00018-008-8357-y 18820996 PMC11131895

[4] Rauh D, Fischer F, Gertz M, Lakshminarasimhan M, Bergbrede T, et al. (2013) An acetylome peptide microarray reveals specificities and deacetylation substrates for all human sirtuin isoforms. Nat Commun 4:2327–43 10.1038/ncomms3327 23995836

[5] Maxwell MM, Tomkinson EM, Nobles J, Wizeman JW, Amore AM, et al. (2011) The Sirtuin 2 microtubule deacetylase is an abundant neuronal protein that accumulates in the aging CNS. Hum Mol Genet 20(20):3986–96. 10.1093/hmg/ddr326 21791548 PMC3203628

[6] Beirowski B, Gustin J, Armour SM, Yamamoto H, North AV, et al. (2011) Sir-two-homolog 2 (Sirt2) modulates peripheral myelination through polarity protein Par-3/atypical protein kinase C (aPKC) signaling. Proc Natl Acad Sci USA 108(43):E952–61. 10.1073/pnas.1104969108 21949390 PMC3203793

[7] Ji S, Doucette JR, Nazarali AJ (2011) Sirt2 is a novel in vivo downstream target of Nkx2.2 and enhances oligodendroglial cell differentiation. J Mol Cell Biol 3(6):351–9. 10.1093/jmcb/mjr009 21669943

[8] Luthi-Carter R, Taylor DM, Pallos J, Lambert E, Amore A, et al. (2010) SIRT2 inhibition achieves neuroprotection by decreasing sterol biosynthesis. Proc Natl Acad Sci USA 107(17):7927–32. 10.1073/pnas.1002924107 20378838 PMC2867924

[9] Porcelli S, Salfi R, Politis A, Atti AR, Albani D, et al. (2013) Association between Sirtuin 2 gene rs10410544 polymorphism and depression in Alzheimer’s disease in two independent European samples. J Neural Transm 120(12):1709–15. 10.1007/s00702-013-1045-6 23712749

[10] Wei W, Xu X, Li H, Y Zhang, Han D, et al. (2014) The SIRT2 Polymorphism rs10410544 and Risk of Alzheimer’s Disease: A Meta-analysis. Neuromolecular Med 16(2):448–56. 10.1007/s12017-014-8291-0 24497179

[11] Outeiro TF, Kontopoulos E, Altmann M, Kufareva I, Strathearn KE, et al. (2007) Sirtuin 2 inhibitors rescue alpha-synuclein-mediated toxicity in models of Parkinson’s disease. Science 317(5837):516–9. 17588900 10.1126/science.1143780

[12] Spires-Jones TL, Fox LM, Rozkalne A, Pitstick R, Carlson GA, et al. (2012) Inhibition of Sirtuin 2 with Sulfobenzoic Acid Derivative AK1 is Non-Toxic and Potentially Neuroprotective in a Mouse Model of Frontotemporal Dementia. Front Pharmacol 12;3:42 10.3389/fphar.2012.00042 22416232 PMC3298895

[13] Taylor DM, Balabadra U, Xiang Z, Woodman B, Meade S, et al. (2011) A brain-permeable small molecule reduces neuronal cholesterol by inhibiting activity of sirtuin 2 deacetylase. ACS Chem Biol. 6(6):540–6. 10.1021/cb100376q 21370928

[14] Chopra V, Quinti L, Kim J, Vollor L, Narayanan KL, et al. (2012) The sirtuin 2 inhibitor AK7 is neuroprotective in Huntington’s disease mouse models. Cell Rep 2(6):1492–7. 10.1016/j.celrep.2012.11.001 23200855 PMC3534897

[15] Dauer W, Przedborski S (2003) Parkinson’s disease: mechanisms and models. Neuron 39(6):889–909. Review. 12971891 10.1016/s0896-6273(03)00568-3

[16] Vila M, Przedborski S (2004) Genetic clues to the pathogenesis of Parkinson’s disease. Nat Med Suppl: S58–62. Review. 15272270 10.1038/nm1068

[17] Körner S, Böselt S, Thau N, Rath KJ, Dengler R, et al. (2013) Differential sirtuin expression patterns in amyotrophic lateral sclerosis (ALS) postmortem tissue: neuroprotective or neurotoxic properties of sirtuins in ALS? Neurodegener Dis 11(3):141–52. 10.1159/000338048 22796962

[18] Narayan N, Lee IH, Borenstein R, Sun J, Wong R, et al. (2012) The NAD-dependent deacetylase SIRT2 is required for programmed necrosis. Nature 492(7428):199–204. 10.1038/nature11700 23201684

[19] Newton K, Hildebrand JM, Shen Z, Rodriguez D, Alvarez-Diaz S, et al. (2014) Is SIRT2 required for necroptosis? Nature 506(7489):E4–6. 10.1038/nature13024 24572428 PMC4005920

[20] Nguyen GT, Gertz M, Steegborn C (2013) Crystal structures of sirt3 complexes with 4’-bromo-resveratrol reveal binding sites and inhibition mechanism. Chem Biol 20(11): 1375–1385. 10.1016/j.chembiol.2013.09.019 24211137

[21] Schutkowski M, Fischer F, Roessler C, Steegborn C (2014) New assays and approaches for discovery and design of Sirtuin modulators. Expert Opin Drug Discov Feb; 9(2):183–99. 10.1517/17460441.2014.875526 24382304

[22] Moniot S. Schutkowski M, Steegborn C (2013) Crystal structure analysis of human Sirt2 and its ADP-ribose complex. J Struct Biol 182(2): 136–143. 10.1016/j.jsb.2013.02.012 23454361

[23] Scholz D, Pöltl D, Genewsky A, Weng M, Waldmann T, et al. (2011) Complete and large-scale generation of post-mitotic neurons from the human LUHMES cell line. J Neurochem 119(5):957–71. 10.1111/j.1471-4159.2011.07255.x 21434924

[24] Schildknecht S, Karreman C, Pöltl D, Efrémova L., Kullmann C, et al. (2013) Generation of genetically-modified human differentiated cells for toxicological tests and the study of neurodegenerative diseases. ALTEX 30(4):427–44. 24173167 10.14573/altex.2013.4.427

[25] Jackson-Lewis V, Przedborski S (2007) Protocol for the MPTP mouse model of Parkinson’s disease. Nat Protoc. 2(1):141–51. 17401348 10.1038/nprot.2006.342

[26] Gurney ME, Pu H, Chiu AY, Dal Canto MC, Polchow CY, et al. (1994) Motor neuron degeneration in mice that express a human Cu,Zn superoxide dismutase mutation. Science 264(5166):1772–5. 8209258 10.1126/science.8209258

[27] Scott S, Kranz JE, Cole J, Lincecum JM, Thompson K, et al. (2008) Design, power, and interpretation of studies in the standard murine model of ALS. Amyotroph Lateral Scler. 9(1):4–15. 10.1080/17482960701856300 18273714

[28] Ludolph AC, Bendotti C, Blaugrund E, Chio A, Greensmith L, et al. (2010) Guidelines for preclinical animal research in ALS/MND: A consensus meeting. Amyotroph Lateral Scler. 11(1–2):38–45. 10.3109/17482960903545334 20184514

[29] Taes I, Timmers M, Hersmus N, Bento-Abreu A, Van Den Bosch L, et al. (2013) Hdac6 deletion delays disease progression in the SOD1G93A mouse model of ALS. Hum. Mol. Genet. 22(9):1783–90. 10.1093/hmg/ddt028 23364049

[30] Dauer W, Przedborski S (2003) Parkinson’s disease: mechanisms and models. Neuron 39(6):889–909. Review. 12971891 10.1016/s0896-6273(03)00568-3

[31] Maries E, Dass B, Collier TJ, Kordower JH, Steece-Collier K (2003) The role of alpha-synuclein in Parkinson’s disease: insights from animal models. Nat Rev. Neurosci. 4(9):727–38. 12951565 10.1038/nrn1199

[32] Bobrowska A, Donmez G, Weiss A, Guarente L, Bates G (2012) SIRT2 ablation has no effect on tubulin acetylation in brain, cholesterol biosynthesis or the progression of Huntington’s disease phenotypes in vivo. PLoS One 7(4):e34805 10.1371/journal.pone.0034805 22511966 PMC3325254

[33] Outeiro TF, Ferreira J (2009) Current and future therapeutic strategies for Parkinson’s disease. Curr Pharm Des 15(34):3968–76. 19751203 10.2174/138161209789649321

[34] Smith JS, Brachmann CB, Celic I, Kenna MA, Muhammad S, et al. (2000) A phylogenetically conserved NAD+-dependent protein deacetylase activity in the Sir2 protein family. Proc Natl Acad Sci USA 97(12):6658–63. 10841563 10.1073/pnas.97.12.6658 PMC18692

[35] Yamagata K, Goto Y, Nishimasu H, Morimoto J, Ishitani R, et al. (2014) Structural basis for potent inhibition of SIRT2 deacetylase by a macrocyclic peptide inducing dynamic structural change. Structure 22(2): 345–352. 10.1016/j.str.2013.12.001 24389023

[36] Lizée G, Aerts JL, Gonzales MI, Chinnasamy N, Morgan RA, et al. (2003) Real-time quantitative reverse transcriptase-polymerase chain reaction as a method for determining lentiviral vector titers and measuring transgene expression. Hum. Gene Ther. 14(6):497–507. 12718761 10.1089/104303403764539387

[37] Kachroo A, Schwarzschild MA (2012) Adenosine A2A receptor gene disruption protects in an α-synuclein model of Parkinson’s disease. Ann. Neurol. 71(2):278–82. 10.1002/ana.22630 22367999 PMC3292742

[38] Chen X, Burdett TC, Desjardins CA, Logan R, Cipriani S, et al. (2013) Disrupted and transgenic urate oxidase alter urate and dopaminergic neurodegeneration. Proc. Natl. Acad. Sci. U S A. 110(1):300–5. 10.1073/pnas.1217296110 23248282 PMC3538212

[39] Wooley CM, Sher RB, Kale A, Frankel WN, Cox GA, et al. (2005) Gait analysis detects early changes in transgenic SOD1(G93A) mice. Muscle Nerve 32(1):43–50. 10.1002/mus.20228 15880561 PMC1350398

[40] Heiman-Patterson TD, Deitch JS, Blankenhorn EP, Erwin KL, Perreault MJ, et al. (2005) Background and gender effects on survival in the TgN(SOD1-G93A)1Gur mouse model of ALS. J. Neurol. Sci. 236(1–2):1–7. 16024047 10.1016/j.jns.2005.02.006

