# Supplementary figures and images for: The Sirtuin-2 Inhibitor AK7 Is Neuroprotective in Models of Parkinson’s Disease but Not Amyotrophic Lateral Sclerosis and Cerebral Ischemia
Source: PLoS One. 2015 Jan 21;10(1):e0116919. doi: 10.1371/journal.pone.0116919 (PMC4301865; doi:10.1371/journal.pone.0116919)

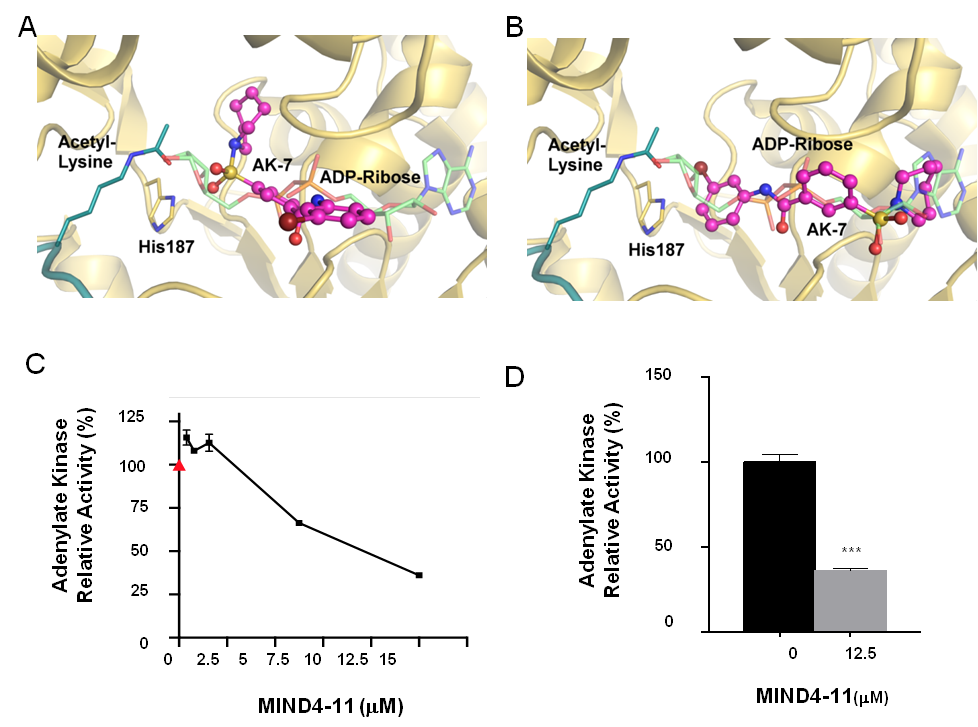

Supplement: S1 Fig — The structure of SIRT2 (PDB ID 3zgv) is shown in gold with the catalytic His187. ADP-ribose (3zgv) and substrate acetylated lysine are shown in light green and deep teal, respectively. AK7 was docked either to Sirt2 in complex with a macrocyclic inhibitor peptide (PDB ID 4l3o) (1A) or to a model of an acetyl-lysine bound SIRT2 generated using 3zgv for the protein and the macrocyclic peptide from 4l3o for the acetyl lysine model (1B). In each cases, the best pose of AK7 (magenta) mainly occupies the NAD+ binding site. (C, D) Protective effects against aSyn toxicity in LUHMES cells of novel SIRT2 inhibitor MIND4-11. MIND4-11 has been identified as selective SIRT2 inhibitor of improved potency (IC50~5mM), albeit lacking brain-permeable property. (C) LUHMES cells were transduced with a lentivirus encoding aSyn. 10 days after differentiation, in the presence of vehicle alone (red, 100% toxicity) or different concentrations of MIND4-11 (black line), media were collected and AK activity was measured. (D) Percentage of AK activity in the presence of 12.5 M of MIND4-11, compared to vehicle-treated cells (*** p< 0.001). (TIF) [file pone.0116919.s001.tif]
